# Supplementary figures and images for: Novel sterol binding domains in bacteria
Source: eLife. 2024 Feb 8;12:RP90696. doi: 10.7554/eLife.90696 (PMC10942540; doi:10.7554/eLife.90696)

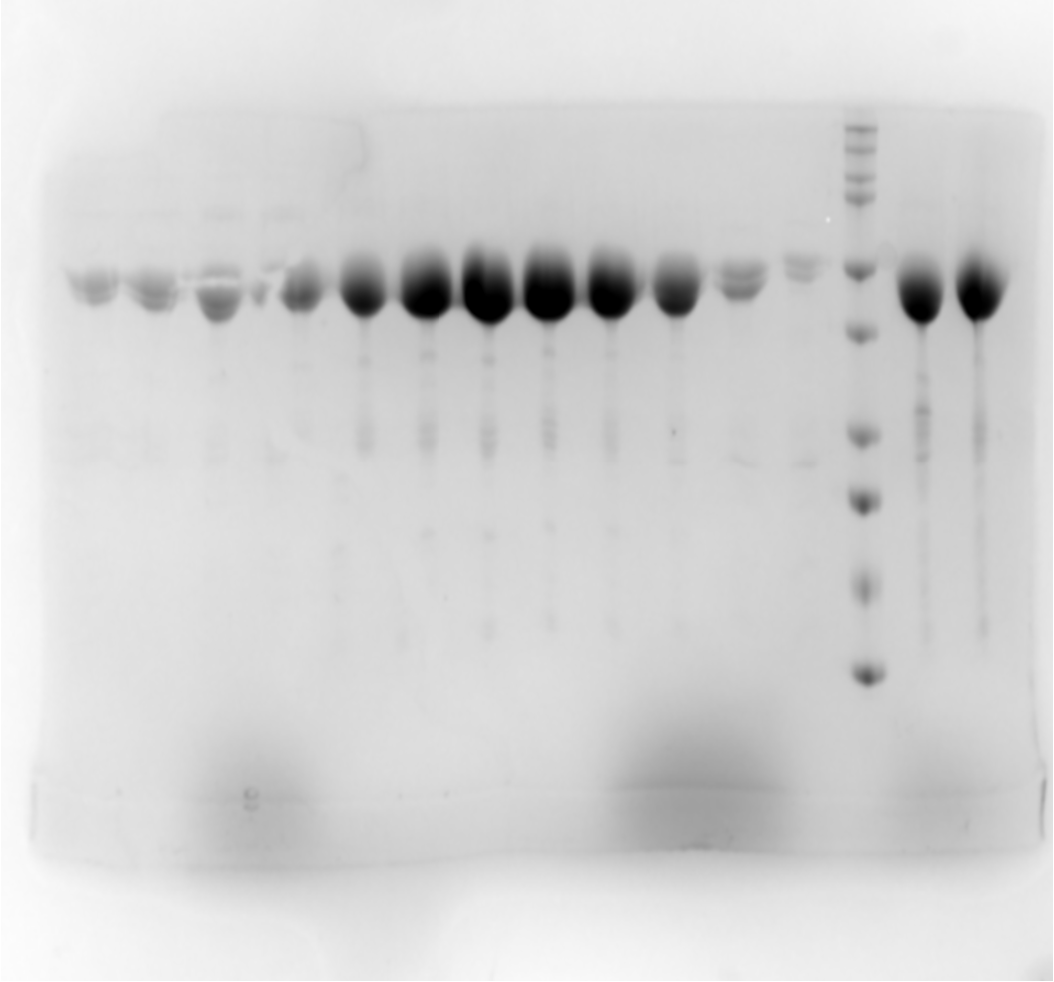

Supplement: Figure 3—figure supplement 1—source data 1. — Original image of Figure 3—figure supplement 1A. [file elife-90696-fig3-figsupp1-data1.zip › Figure 3- figure supplement 1 - source data 1/BstA purification gel.png]

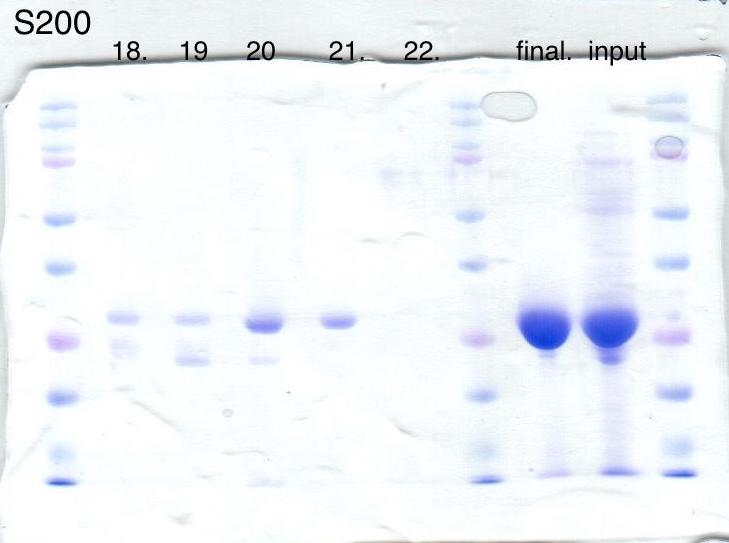

Supplement: Figure 3—figure supplement 1—source data 3. — Original image of Figure 3—figure supplement 1A. [file elife-90696-fig3-figsupp1-data3.zip › Figure 3- figure supplement 1 - source data 3/20191230 purification of BstB.jpg]

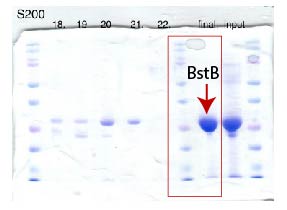

Supplement: Figure 3—figure supplement 1—source data 4. — Original image of Figure 3—figure supplement 1A marked to show the band depicted in the figure. [file elife-90696-fig3-figsupp1-data4.zip › Figure 3- figure supplement 1 - source data 4/BstB purification gel-marked.jpg]

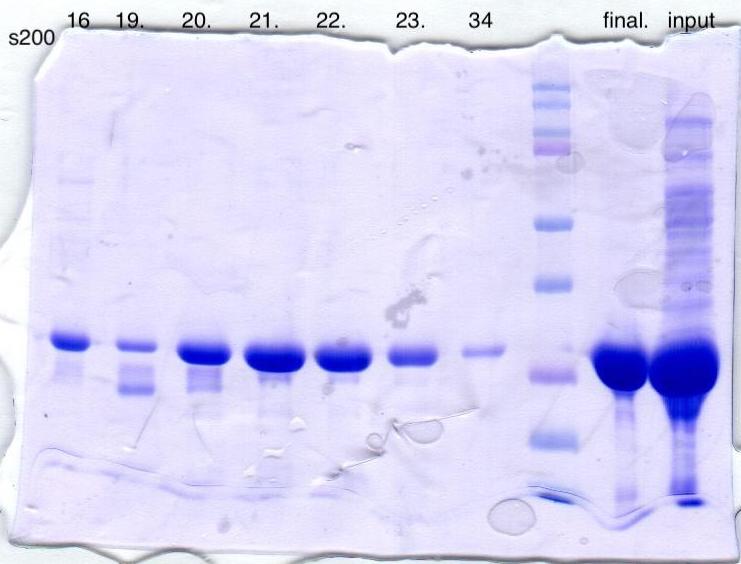

Supplement: Figure 3—figure supplement 1—source data 5. — Original image of Figure 3—figure supplement 1A. [file elife-90696-fig3-figsupp1-data5.zip › Figure 3- figure supplement 1 - source data 5/20200102 purification of BstC.jpg]

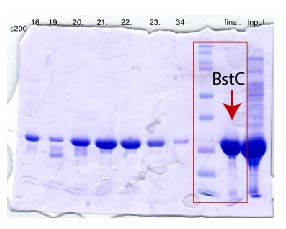

Supplement: Figure 3—figure supplement 1—source data 6. — Original image of Figure 3—figure supplement 1A marked to show the band depicted in the figure. [file elife-90696-fig3-figsupp1-data6.zip › Figure 3- figure supplement 1 - source data 6/BstC purification gel-marked.jpg]

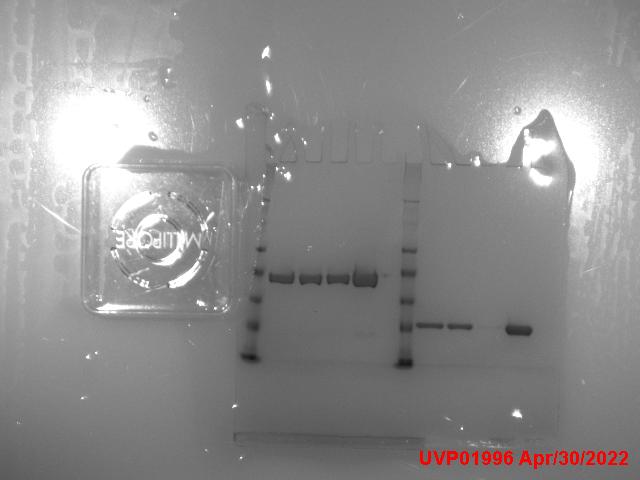

Supplement: Figure 3—figure supplement 1—source data 7. — Original image of Figure 3—figure supplement 1C. [file elife-90696-fig3-figsupp1-data7.zip › Figure 3- figure supplement 1 - source data 7/BstA and BstB pull downs2_2_Apr302022.jpg]

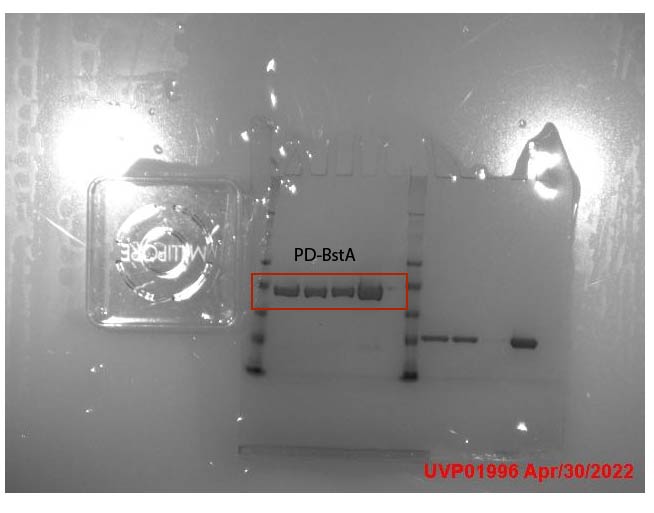

Supplement: Figure 3—figure supplement 1—source data 8. — Original image of Figure 3—figure supplement 1C marked to show the bands depicted in the figure. [file elife-90696-fig3-figsupp1-data8.zip › Figure 3- figure supplement 1 - source data 8/PD-BstA_pulldown_marked.jpg]

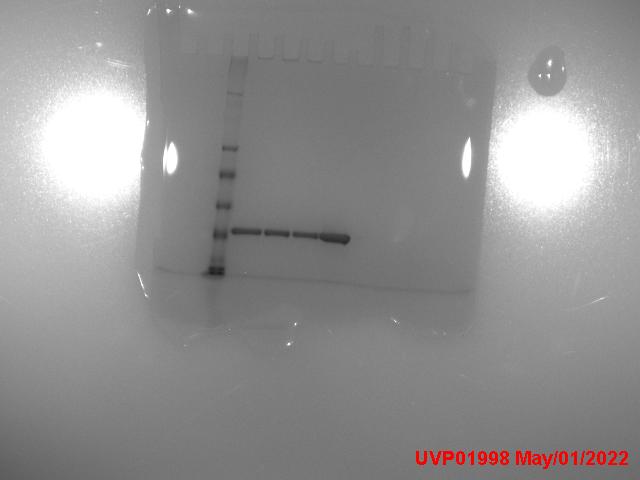

Supplement: Figure 3—figure supplement 1—source data 9. — Original image of Figure 3—figure supplement 1C. [file elife-90696-fig3-figsupp1-data9.zip › Figure 3- figure supplement 1 - source data 9/BstB pull down_2_May12022.jpg]

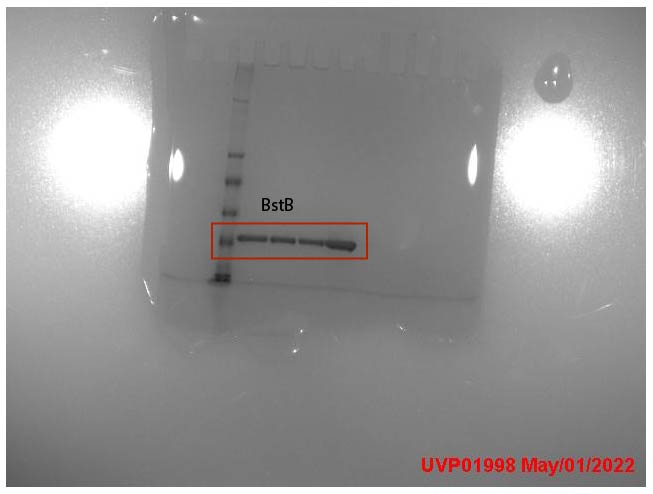

Supplement: Figure 3—figure supplement 1—source data 10. — Original image of Figure 3—figure supplement 1C marked to show the bands depicted in the figure. [file elife-90696-fig3-figsupp1-data10.zip › Figure 3- figure supplement 1 - source data 10/BstB_pulldown_marked.jpg]

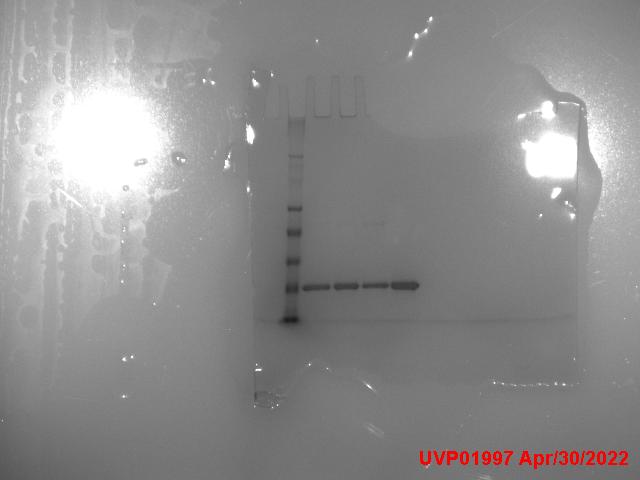

Supplement: Figure 3—figure supplement 1—source data 11. — Original image of Figure 3—figure supplement 1C. [file elife-90696-fig3-figsupp1-data11.zip › Figure 3- figure supplement 1 - source data 11/BstC pull down_2_Apr302022.jpg]

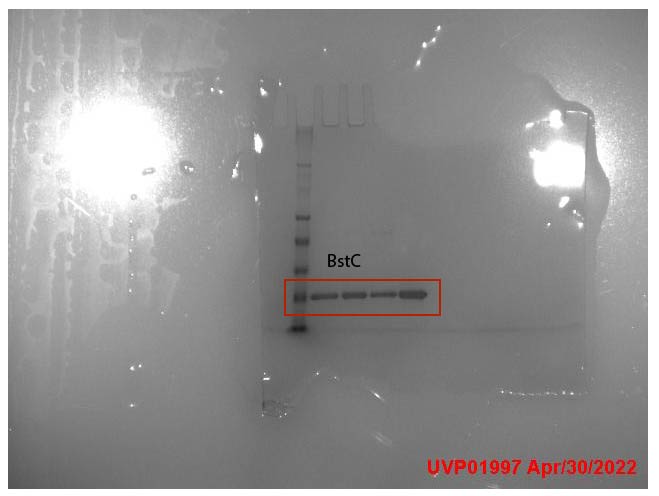

Supplement: Figure 3—figure supplement 1—source data 12. — Original image of Figure 3—figure supplement 1C marked to show the bands depicted in the figure. [file elife-90696-fig3-figsupp1-data12.zip › Figure 3- figure supplement 1 - source data 12/BstC_pulldown_marked.jpg]
